# Banana ripening control: a non-canonical F-box protein links ethylene and ABA signaling

**DOI:** 10.1093/plphys/kiab560

**Published:** 2022-02-03

**Authors:** Madeleine Seale

**Affiliations:** Department of Plant Sciences, University of Oxford, South Parks Road, Oxford, OX1 3RB, UK

From field to consumer, global loss and wastage of fruit and vegetables are thought to be around 37% (Spang et al., 2019). Controlling ripening and limiting damage to fruits are important strategies for minimizing these losses. Two papers published in *Plant Physiology* this month have focused on banana (*Musa* species) fruit ripening, investigating both transcriptional and post-translational regulation of ripening (Song et al., 2021; Wu et al., 2021). One of these investigated the interplay between ethylene and abscisic acid (ABA) signaling, establishing that a banana (*Musa* ABB Pisang Awak) ethylene-signaling related F-box protein EIN3-BINDING F BOX PROTEIN 1 (EBF1) interacts with the transcription factor ABSCISIC ACID-INSENSITIVE 5 (ABI5)-LIKE to soften fruit by activating starch and cell wall degradation enzymes (Song et al., 2021). Intriguingly, MaEBF1 does not appear to behave as a classic F-box protein in this context as no ubiquitination of targets could be detected. 

In recent decades, fruit development and ripening research has focused heavily on tomato (*Solanum lycopersicum*) as a model system. Despite many commonalities between tomato ripening and other species, fruits have evolved independently multiple times across angiosperms. Many species, including tomato and banana, undergo an ethylene burst and enhanced respiratory rate at the onset of ripening and are known as climacteric fruits. While the same hormones and many genetic regulators have been either retained or repeatedly recruited for ripening control, convergent evolution means that precise mechanisms are not necessarily identical across species (Lü et al., 2018).

Banana fruit ripening appears particularly complex in comparison to other climacteric fruits, involving at least two interlocking regulatory loops involving ethylene and MCM1-AGAMOUS-DEFICIENS-SERUM RESPONSE FACTOR (MADS) and NAM/ATAF/CUC (NAC) transcription factors to activate ripening genes (Lü et al., 2018). ABA also works alongside ethylene to promote the proper progression of ripening in climacteric fruits and has been specifically associated with fruit softening in both banana and tomato (Forlani et al., 2019).

Song et al. (2021) used chilling storage treatments to investigate the dynamic changes to hormone signaling and fruit ripening progression. Storage at 11°C delayed fruit ripening but a prolonged reduction in temperature to 7°C resulted in damage and prevented fruits from ripening at all, even when treated with an ethylene precursor that normally stimulates ripening.

**Figure 1:**
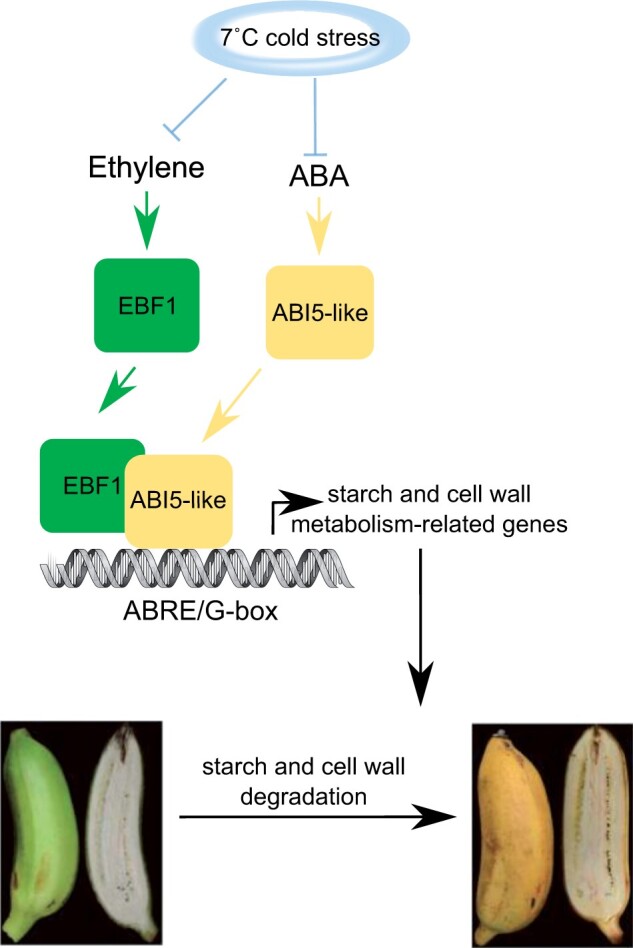
Schematic illustrating the model by which ethylene and ABA regulate fruit ripening via EBF1 and ABI5-like. Adapted from Song et al. (2021). Expression of *MaEBF1* and *MaABI5-like* increases during ripening but can be suppressed by chilling. MaEBF1- and MaABI5-like proteins physically interact and MaABI5-like can bind to ABRE elements in promoter regions. The proteins increase transcription of starch and cell wall degradation enzymes to promote fruit ripening.

These chilling treatments corresponded to reduced expression of starch and cell wall degradation enzymes, corresponding with reduced expression of *MaEBF1* and *MaABI5-like*. The authors went on to establish that MaEBF1 and MaABI5-like proteins physically interact in several protein interaction assays. When MaABI5-like or MaEBF1 were each transiently expressed in *Nicotiana benthamiana*, they induced luciferase expression of several starch and cell wall degradation promoters that had been fused to dual-luciferase reporters. Luciferase signals were further enhanced when both MaABI5-like and MaEBF1 were co-expressed. These data indicate that both proteins work together to promote the expression of fruit ripening genes (Figure  1).

As *EBF1* homologs in other species ubiquitinate proteins to stimulate their degradation, the authors investigated whether this occurred in banana fruit ripening or chilling. While Arabidopsis (*Arabidopsis thaliana*) AtEBF1 was able to ubiquitinate the ethylene signaling protein ETHYLENE INSENSITIVE 3 (AtEIN3), MaEBF1 did not ubiquitinate either MaABI5-like or MaEIN3-LIKE banana proteins. In fact, concomitantly with MaEBF1, protein levels of both MaABI5-like and MaEILs increased during fruit ripening, further confirming that targeted degradation via MaEBF1 does not occur. This strongly suggests that MaEBF1 acts in a different manner to the EBFs of eudicot species studied previously. The work presented builds on an emerging picture that mechanisms controlling ripening progression vary among climacteric fruits despite superficial similarities. For example, banana appears to have additional ethylene synthesis mechanisms, compared to tomato, that are insensitive to pharmacological inhibition of ethylene perception (Golding et al., 1998; Pathak et al., 2003).

Despite these differences, overexpression of MaEBF1 and MaABI5-like in tomato resulted in enhanced fruit ripening and softening. This contrasts with overexpression of native tomato EBFs, which results in reduced sensitivity to ethylene and inhibition of ripening (Deng et al., 2018; Guo et al., 2018). It appears that the banana MaEBF1 is not a true ortholog of the tomato EBFs but that the EBF1-ABI5 module can function alongside the existing tomato ripening machinery to degrade starch and cellulose within the fruit.

*ABI5-like* is a basic leucine zipper (bZIP) transcription factor, and homologs in many species are involved in ABA signaling. The data presented here indicate that MaEBF1 functions as a transcriptional coactivator in conjunction with ABI5-like to mediate the effects of both ethylene and ABA in fruit ripening at a transcriptional level (Figure  1). Interestingly, Wu et al. (2021) identified another bZIP transcription factor, MabZIP21, with a key role in accelerating banana fruit ripening. Though MaABI5-like is not post-translationally regulated by MaEBF1, MabZIP21 appears to be post-translationally regulated through phosphorylation by MITOGEN ACTIVATED PROTEIN KINASE 6-3 (MaMPK-3). It will be interesting to investigate whether there is communication between these two signaling modules or whether they act in parallel.

The interplay between hormones and transcription factors is becoming clearer for banana fruit ripening and is starting to provide some explanations for species-specific developmental programs. It is clear that evolution has used similar molecular tools to regulate fruit ripening across species but has fine-tuned those mechanisms in a variety of ways to provide the diverse fruits and ripening dynamics observed among angiosperms.

*Conflict of* *interest statement*. No conflict of interest.
